# Diffusion Tensor Imaging and Tractography Utilized in the Resection of a Midbrain Cavernous Malformation

**DOI:** 10.31486/toj.19.0017

**Published:** 2020

**Authors:** Joseph Saliba, Andrew Steven, John Franklin Berry, Edison P. Valle-Giler

**Affiliations:** ^1^ Department of Radiology, Ochsner Clinic Foundation, New Orleans, LA; ^2^ Department of Neurosurgery, Ochsner Clinic Foundation, New Orleans, LA; ^3^ The University of Queensland Faculty of Medicine, Ochsner Clinical School, New Orleans, LA

**Keywords:** *Diffusion magnetic resonance imaging*, *diffusion tensor imaging*, *hemangioma–cavernous*

## Abstract

**Background:** Diffusion tensor imaging (DTI) is a magnetic resonance–based imaging technique that can provide important information about the underlying structure and integrity of the white matter in the brain. Tractography, a DTI postprocessing technique, can provide a detailed model of individual white matter fiber tracts. Knowledge of these tracts may be beneficial in the surgical planning and execution for neurosurgical patients.

**Case Report:** We review the basic principles behind DTI and present an illustrative case in which DTI was used to delineate the relationship of eloquent white matter tracts to a cavernous malformation in a patient undergoing resection.

**Conclusion:** The use of DTI during preoperative planning allows the neurosurgeon to understand if a lesion is disrupting, infiltrating, or altering the course of local white matter tracts. With the combined use of DTI and intraoperative neuronavigation, the neurosurgeon can better identify and avoid white matter tracts, not only in the local area of resection but also during approach to the lesion, thereby reducing the risk of damage to vital cortical pathways and subsequent functional impairment.

## INTRODUCTION

Diffusion tensor imaging (DTI) is a magnetic resonance–based imaging technique that can provide important information about the underlying structure and integrity of the white matter in the brain.^1,2^ Tractography, a DTI postprocessing technique, can provide a detailed model of individual white matter fiber tracts.^3^ Knowledge of these tracts may be beneficial in the surgical planning and execution for neurosurgical patients.^4^ We review the basic principles behind DTI and present an illustrative case in which DTI was used to delineate the relationship of eloquent white matter tracts to a cavernous malformation in a patient undergoing resection.

## CASE REPORT

**Figure 1.**
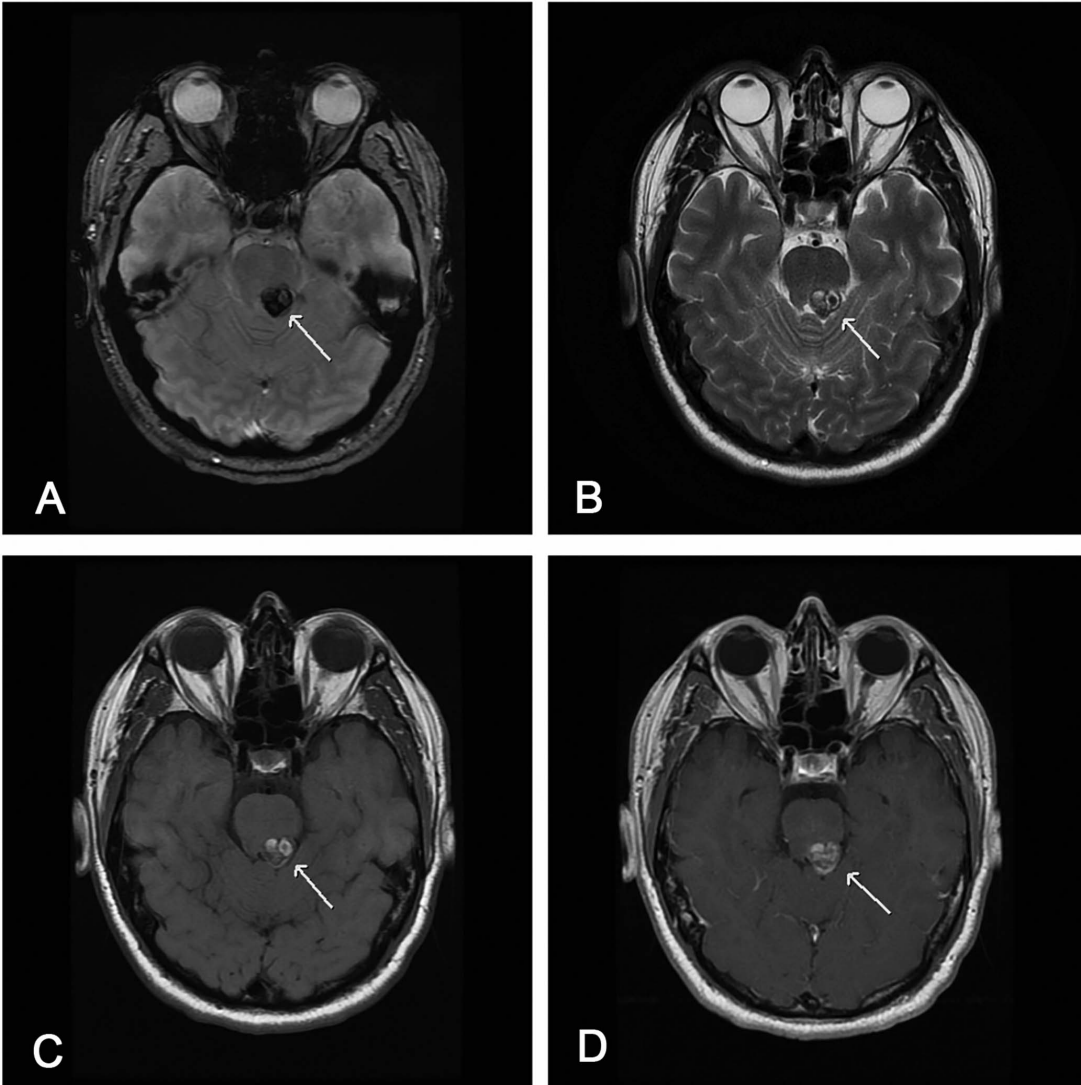
**Dorsal midbrain cavernous malformation demonstrating classic popcorn appearance. Axial T2 gradient echo image (A) shows prominent susceptibility artifact indicating blood products (arrow). Axial T2-weighted image (B) depicts heterogeneous signal centrally with hypointense hemosiderin rim (arrow). No surrounding edema. T1-weighted images before (C) and after (D) intravenous contrast administration show minimal postcontrast enhancement of the lesion (arrows).**

A 51-year-old male presented with diplopia and fourth cranial nerve palsy. He also reported the feeling of “being wet” over the entire right side of his body. Magnetic resonance imaging (MRI) revealed a hemorrhagic lesion within the dorsal left midbrain, exhibiting classic imaging characteristics of a cavernous malformation (Figure 1). Initially, invasive management was deferred, and follow-up MRI was planned. Upon return to clinic at 7 months, the patient reported resolution of his diplopia but persistence of his sensory abnormality and new balance problems. Repeat MRI showed enlargement of the cavernous malformation.

**Figure 2.**
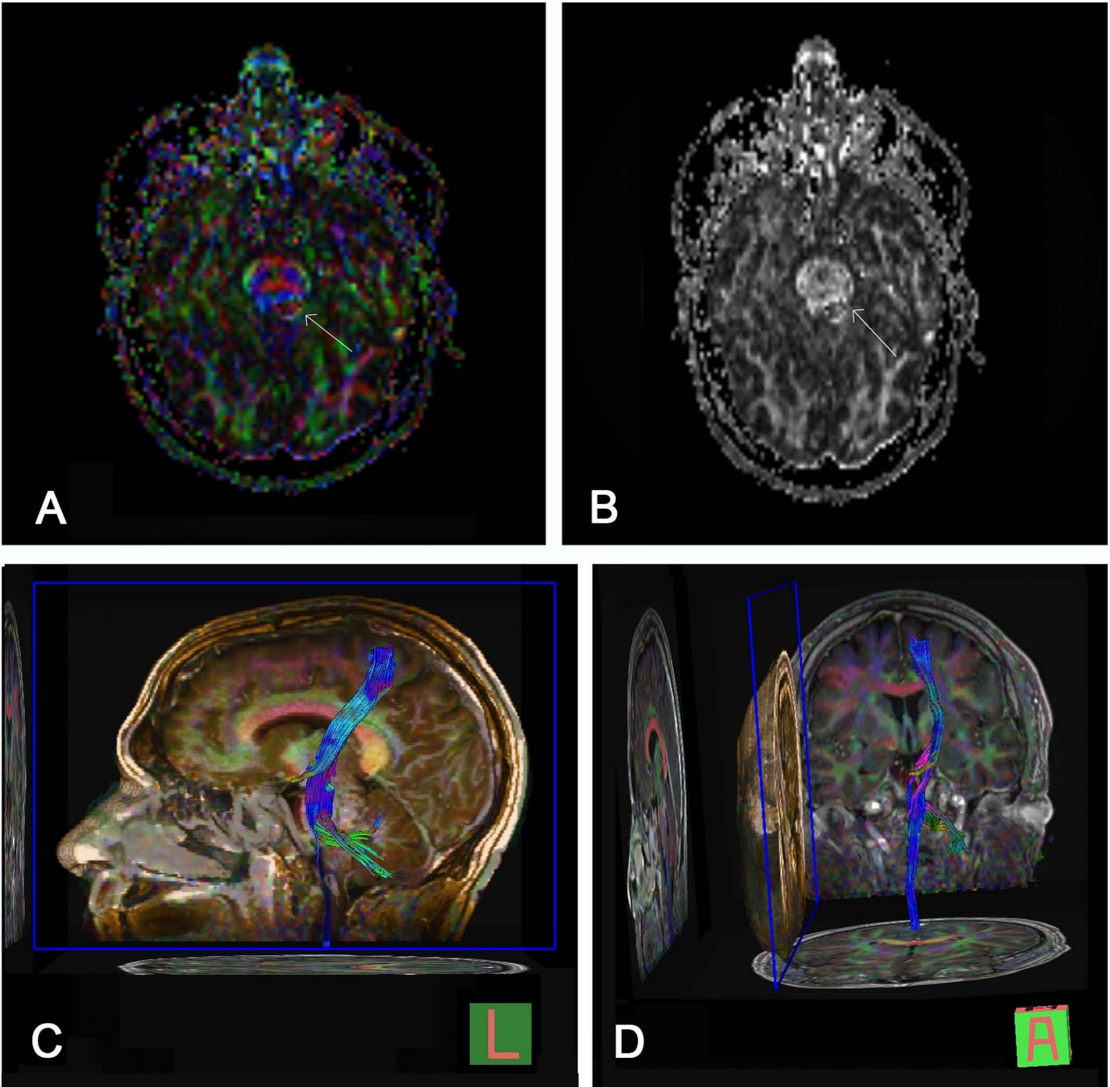
**Diffusion tensor imaging with tractography fractional anisotropy maps with (A) and without (B) directional color information showing effacement of the superior cerebellar peduncle and mild displacement of the medial lemniscus (arrows). Transverse pontine fibers and pyramidal tract are intact anterior to the lesion. Three-dimensional sagittal (C) and coronal (D) tractograms of the left corticospinal tract demonstrate the relationship of the cavernous malformation to traversing white matter bundles.**

Because of the progressive symptoms and lesion enlargement, resection was planned. Preoperative MRI with DTI showed effacement of the left superior cerebellar peduncle and subtle displacement of the medial lemniscus. The transverse pontine fibers and pyramidal tract were intact (Figure 2).

The cavernous malformation was successfully resected using tractography for preprocedural planning and neuro-navigation in the operative suite. The patient tolerated the procedure well, without apparent complications. At his 6-week follow-up evaluation, he had only mild residual left-sided fourth nerve palsy with minimal double vision.

## DISCUSSION

MRI diffusion imaging is based on the spatial mapping of the random motion of water molecules known as Brownian motion. While the initial application of diffusion imaging focused on stroke detection, imaging techniques have progressed to include a wide range of clinical applications. DTI, a more advanced application than basic diffusion-weighted imaging, interrogates the directionality of water diffusion. DTI can provide unique information about the structure of the brain, and its clinical use is increasing.^5-7^

The diffusion of water molecules within organized tissues is directionally dependent, a property known as anisotropy. By using the appropriate magnetic field gradient, diffusion imaging can be sensitized to the motion of water molecules aligned with the gradient. By changing the direction of these gradients, the direction of maximum diffusivity can be determined.^5^ This information is particularly useful in the evaluation of white matter tracts where the axonal membrane and myelin sheath limit the free diffusion of water.^2^ Within these tracts, the direction of maximum diffusivity has been demonstrated to correspond to tract fiber orientation.^7^ This information is quantified in a 3-dimensional (3-D) mathematical model known as a diffusion tensor that contains information regarding the orientation and degree of anisotropy to produce an effective diffusion ellipsoid for each voxel in which the diameter in any direction coincides with the degree of diffusivity in that direction.^5,7^ Color maps can be constructed voxel by voxel, using red, green, and blue color channels to represent the direction of maximal diffusivity tracts coursing in transverse, anteroposterior, and superoinferior directions, respectively (Figure 2).

While multiple DTI metrics are available—such as axial diffusivity, radial diffusivity, and tumor infiltration index—the most commonly used is fractional anisotropy (FA), which quantifies the extent of directionality on a scale of 0 (isotropy) to 1 (maximum anisotropy). FA values may be affected in several processes that alter the normal white matter structure such as edema, trauma, demyelination, and tumor infiltration.^6,7^ FA values are used to modulate the signal intensity of DTI maps, creating maps that display both the direction of white matter fibers and the degree of anisotropy. Tractography is a postacquisition modeling technique that uses the diffusion tensor data to produce 3-D models of white matter fiber tracts.^3^ Several methods have been described to create these models that were concordant with known white matter fiber tract anatomy.^1,8-10^

A primary goal of neurosurgical treatment is preserving cerebral function while maximizing lesion resection. Achieving this goal requires knowledge about lesion extent and association with surrounding functional structures, including eloquent cortex and white matter tracts. While functional MRI is available for mapping eloquent cerebral cortex that may be affected by a tumor, it does not provide information about the involvement of subjacent white matter tracts. DTI, however, is an exceptional tool for the visualization of white matter tracts to aid in the planning of surgical intervention.^4^ Alterations in white matter produced by intracranial masses and tumors may present with patterns on DTI maps that can be broadly categorized into 2 categories: (1) deviation refers to tracts that are displaced by tumor bulk or edema but remain intact, and (2) infiltration refers to tracts that have been completely disrupted and are no longer identifiable on color maps.^7,11^

DTI tractography thus provides a useful tool for the neurosurgeon in pursuing maximal lesion resection with minimal normal tissue disruption, and subsequently, minimal disruption of normal neurologic function. The use of DTI during preoperative planning allows the neurosurgeon to understand if a lesion is disrupting, infiltrating, or altering the course of local white matter tracts. In the case of deep-seated subcortical lesions, superficial tracts that may be encountered during the approach to the lesion can be visualized,^12^ allowing the surgeon to choose a safe approach to the lesion and make appropriate modifications to standard surgical approaches. With the combined use of DTI and intraoperative neuronavigation, the neurosurgeon can better identify and avoid white matter tracts, not only in the local area of resection but also during approach to the lesion, thereby reducing the risk of damage to vital cortical pathways and subsequent functional impairment.

## CONCLUSION

DTI provides considerable information about the underlying white matter structure of the brain, including valuable information about the relationship of these tracts to local lesions, as well as the lesion itself. These data can provide unique guidance in the planning and execution of neurosurgical intervention.

## Notes

This article meets the Accreditation Council for Graduate Medical Education and the American Board of Medical Specialties Maintenance of Certification competencies for Patient Care and Medical Knowledge.
